# B7-H3 in glioblastoma and beyond: significance and therapeutic strategies

**DOI:** 10.3389/fimmu.2024.1495283

**Published:** 2024-11-25

**Authors:** Davor Babič, Ivana Jovčevska, Alja Zottel

**Affiliations:** Centre for Functional Genomics and Bio-Chips, Institute of Biochemistry and Molecular Genetics, Faculty of Medicine, University of Ljubljana, Ljubljana, Slovenia

**Keywords:** glioblastoma, B7-H3, CD276, signaling pathways, immune checkpoints, immunotherapy

## Abstract

Cancer has emerged as the second most prevalent disease and the leading cause of death, claiming the lives of 10 million individuals each year. The predominant varieties of cancer encompass breast, lung, colon, rectal, and prostate cancers. Among the more aggressive malignancies is glioblastoma, categorized as WHO stage 4 brain cancer. Following diagnosis, the typical life expectancy ranges from 12 to 15 months, as current established treatments like surgical intervention, radiotherapy, and chemotherapy using temozolomide exhibit limited effectiveness. Beyond conventional approaches, the exploration of immunotherapy for glioblastoma treatment is underway. A methodology involves CAR-T cells, monoclonal antibodies, ADCC and nanobodies sourced from camelids. Immunotherapy’s recent focal point is the cellular ligand B7-H3, notably abundant in tumor cells while either scarce or absent in normal ones. Its expression elevates with cancer progression and serves as a promising prognostic marker. In this article, we delve into the essence of B7-H3, elucidating its function and involvement in signaling pathways. We delineate the receptors it binds to and its significance in glioblastoma and other cancer types. Lastly, we examine its role in immunotherapy and the utilization of nanobodies in this domain.

## 1 Introduction

Cancer is a significant health issue, being a disease of considerable concern. In 2020, it was the second leading cause of death in the United States, with 602,350 deaths, following heart disease, which claimed 696,962 lives. It is noteworthy that cancer stands as a primary cause of death among women aged 40-59 years and both genders within the 60-79 year age range. While there has been a reduction in mortality rates for certain cancers such as leukemia, melanoma, and kidney cancer, it is important to mention that other cancers like glioblastoma continue to sustain their high fatality rates (1).

Glioblastoma is the most common and malignant primary brain cancer. The World Health Organization (WHO) classifies glioblastoma as grade IV brain tumor. Currently patients with glioblastoma first undergo surgical resection which is followed by radiotherapy and chemotherapy with temozolomide (2). Despite the combinatorial treatment, 5-year survival rate is less than 10% (3). Low survival rate of glioblastoma patients can be attributed to several factors. First, glioblastoma is a very heterogeneous cancer with different mutations (4). Second, it is very difficult to resect tumor completely due to the possibility of damaging nearby brain tissue (5). Third, tumor microenvironment (TME) suppresses the immune system (6). Fourth, blood-brain barrier (BBB) limits drug delivery to tumor (7). A recent publication by Lizhi Pang et al. describes a significant role of TME in glioblastoma. However, understanding the heterogeneity of glioblastoma remains challenging due to methodological limitations. Artificial intelligence (AI) could offer a step forward in uncovering brain tumor heterogeneity with introduction of different data analysis and predictive models. AI can significantly boost speed with analysis of large date such as single cell and protein interactions. Machine learning can predict different genetic drivers, tumor progression and patient survival. Such an approach could enable personalized and precise interventions, leading to reliable outcomes in scientific and medical advancements (8).

Recently, new drugs and methods of treatment have been developing including immunotherapy. Different approaches of immunotherapy have been developed including immune checkpoint inhibitors, oncolytic virus therapies, cancer vaccines, adaptive cell transfer and cytokine therapies. Immunotherapy has been to some extent successfully implemented in treatments of several cancers (9). In the case of glioblastoma, researchers have so far been able to reach phase 3 of clinical trials using immunotherapies where no significant improvements in terms of median overall survival have been shown (10–13).

New methods based on nanotechnology are developing including nanobodies. Nanobodies have several advantages over conventional antibodies. Some studies indicate that nanobodies might diffuse through BBB (14) and TME, giving hope for increased immunotherapeutic efficacy (15). One of the potential targets is ligand B7-H3, due to its high expression on different cancers and correlation to poor patient prognosis (16, 17). Several different approaches targeting B7-H3 are being investigated, including chimeric antigen receptor (CAR) T-cells as the most studied approach. In this review we focus our research on B7-H3 and its immunotherapy considering glioblastomas. Several other cancers will also be described. At last, nanobodies and their therapeutic use in immunotherapy will be presented including latest research on nanobodies in glioblastoma and B7-H3.

## 2 B7 homolog 3 (B7-H3)

B7 homolog 3 (B7-H3) is an immunoregulatory checkpoint glycoprotein and a member of B7 family regulatory ligands, responsible for regulating T cell lymphocytes and tumorigenesis. Members of this family are cluster of differentiation 80 (CD80 or B7-1), CD86 (B7-2), CD274 (B7-H1 or PD-L1), PDCD1LG2 (PD-L2), ICOSLG (B7-H2), CD276 (B7-H3), VTCN1 (B7-H4), VSIR (B7-H5), NCR3LG1 (B7-H6), HHLA2 (B7-H7) and ILDR2 (18). Members of B7 family are a group of proteins important for immune homeostasis. They are primarily involved in costimulatory and coinhibitory signals that modulate T-cell activation, proliferation, and function. The interaction between B7 proteins and their receptors can either suppress or enhance immune response, making them critical in the context of cancer or autoimmune diseases. B7-1, B7-2 and B7-H2 are important for regulating immune tolerance and naïve T cell activation. Meanwhile PD-L1, PD-L2, HHLA2, B7-H3 and B7-H4 are responsible for regulating immune response in peripherial tissues (19). B7-H3 has called for attention due to its high expression on cancer cells and lower or none in normal cells. Additionally, its high expression in cancer cells is correlated with poor prognosis (20–23). The expression of B7-H3 is higher in glioblastoma, while it shows minimal presence in normal tissues, making it an interesting therapeutic target. Some FDA approved drugs targeting B7-H3 such as ^131^I Omburtamab for neuroblastoma, Enoblituzumab for prostate cancer, and GSK5764227 (HS-20093) for small-cell lung cancer, highlights the progress in this area. The development of B7-H3 targeting drugs is advancing, and there is great potential to investigate their therapeutic effects in glioblastoma, a field where the full therapeutic potential has not yet been elucidated (24).

B7-H3 is a type I transmembrane glycoprotein with an extracellular domain containing IgV-like and IgC-like domain, transmembrane domain and short intracellular domain containing short cytoplasmic tail. Two isoforms of B7-H3 are discovered in humans, 2IgB7-H3 (25) and 4IgB7-H3 (26, 27). B7-H3 is located at 15q24.1. The size of 4IgB7-H3 is 534 amino acids and encode a 110 kDa protein (26), while 2IgB7-H3 is 306 amino acids and encodes 45-66 kDa (25, 28). In non-cancerous brain tissues of patients only 2IgB7-H3 was present, while in glioblastoma tissues 2IgB7-H3 expression was decreased and 4IgB7-H3 expression was expressed. Interestingly 2IgB7-H3 expression was higher in glioblastoma recurrences compared to newly diagnosed glioblastoma (20). Soluble B7-H3 (sB7-H3) is 16.5 kDa and is detected in serum levels of healthy individuals. Monocyte, dendritic cells, activated T cells and some cancer cell lines (ovarian, breast, lung) release sB7-H3. Evidence indicate that sB7-H3 is released with a help of matrix metalloproteinase (MMP) (29).

## 3 B7-H3 receptors

B7-H3 is known for its dual role in physiology, possessing stimulatory and inhibitory function in cancer development (30). Many factors influence this type of action. It may depend on cell type on which it is expressed, cell receptor (31), the isoform being present (32), posttranscriptional regulation (33), epigenetic and posttranslational modifications (21, 34).

### 3.1 TLT-2

The first possible receptor identified for 2IgB7-H3 was TLT-2, which was found to bind fusion protein of murine B7-H3 and human IgG1 (B7-H3Ig) in murine T cells. The interaction with TLT-2 resulted in enhanced T-cell activation, especially CD8+ T cells correspondingly enhancing production of IFN-γ and IL-2. However, it should be considered that fusion protein B7-H3-Ig and innate membrane B7-H3 are different, the same as TLT-2 expression in transfected cells and TLT-2 normally found on cells (31). Studies show contradictory results, while some claim that both 2IgB7-H3 and 4IgB7-H3 inhibit T cell activation (35), others show the contrary (31). Additionally, Leitner et al. (35) showed that TLT-2 on human T cells does not serve as a receptor for neither 2IgB7-H3 or 4IgB7-H3 even though there is some kind of receptor on T cells that interact with B7-H3. Even though Hashiguchi et al. (31) found interaction with TLT-2 and B7-H3Ig in murine system, Leitner et al. (35) and Yan et al. (36) were not successful in reproducing the results (35). The existence of TLT-2 remains controversial, and a need of better understanding is crucial to elucidate described contradictories. It should be considered that although the same methods were used, fusion protein construct, transduction efficiency and cellular background can result in different outcomes.

### 3.2 Interleukin 20 receptor subunit alpha (IL20RA)

Using new interactome technique, Husein et al. identified another possible receptor – the interleukin 20 receptor subunit alpha (IL20RA) (37). IL20RA is a subunit of the IL20 along with IL20RB. Upon binding of its ligand, it can form heterodimer with IL20RB. IL20RA has many ligands including IL-19, IL-20, and IL-24 (38, 39). IL20RA is expressed in skin, ovary, placenta, lungs and testes (38). IL20RA is expressed in human skin, higher in adults compared to children skin (40). It is involved in many cancers by regulating signaling pathways. Overexpression of IL20RA was found in colorectal cancer (CRC) and was also associated with greater tumor diameter and poor prognosis (41). Knockdown of IL20RA in CRC cell lines downregulates Janus kinase 1 (JAK1) and signal transducer and activator of transcription 3 (STAT3) and consistently suppress tumor growth (42). On the other hand, activation of IL20RA in ovarian cancer cells, when they disseminate into peritoneal cavity, results in polarization of macrophages into anti-tumor M1 subtype (43). They also showed expression of IL20RA and IL20RB in human brain micro vessels (44). There are currently no findings connecting IL20RA and glioblastoma.

Using Conditioned Media AlphaScreen technology, IL20RA was confirmed as an interacting partner of recombinant B7-H3 protein. B7-H3 was expressed as biotinylated Avi-tagged extracellular domain using *E. coli*. Additionally, binding of recombinant B7-H3 on cells transiently expressing IL20RA was analyzed with immunofluorescence. Results confirm binding of B7-H3 only on cells expressing IL20RA. This is a promising finding of another B7-H3 receptor but should be confirmed with other studies (37).

### 3.3 Angio-associated migratory cell protein (AAMP)

Recently a new potential B7-H3 receptor has been identified adding another piece to a puzzle of dual B7-H3 functioning. Angio-associated migratory cell protein (AAMP) plays a role in cell migration and regulates angiogenesis (45). AAMP has also role in different cancers including being overexpressed in breast cancer (46) and gastrointestinal stromal tumors (47). Furthermore AAMP is associated with poor clinical outcomes in CRC patients promoting invasion and migration both *in vitro* and *in vivo* (48). Using yeast two-hybrid screening (Y2H) and mass spectrometry phosphorylation screen, 17 potential interacting partners on natural killer (NK) cells were identified. After bimolecular fluorescent complementation (BiFC) assay, 4 candidate genes were revealed: cluster of differentiation 164 (CD164), AAMP, receptor-type tyrosine-protein phosphatase alpha (PTPRA) and SLAM Family Member 7 (SLAMF7). After that endogenously binding partners were further accessed using co-immunoprecipitation (co-IP), results confirmed AAMP as the only interaction partner. Additionally, B7-H3 interaction with AAMP knockdown and control Jurkat cell lines was analyzed. The highest concentration of B7-H3 used equally inhibited the proliferation of both cell lines, while the lower concentration of B7-H3 inhibited the knockdown of Jurkat cells more, indicating that AAMP has at least a partial interaction with B7-H3 and reduces the antiproliferative effect of B7-H3 to a limited extent. To further validate the finding, they used multivariate expression of these two genes in glioblastoma patients and showed a positive correlation in expression. Also, poor prognosis is only observed for patients with high AAMP and B7-H3 expression. Tumors can glycosylate proteins and change their interaction with receptors additionally adding another level of complexity. CD164, PTPRA and SLAMF7 could not be verified further due to shorter interaction time, poorly evaluated antibodies or Y2H false positives (49).

## 4 B7-H3 posttranscriptional regulation

miRNAs represent a very unique group of small RNAs responsible for regulating gene expression. Deregulation of miRNAs can lead to the development of various cancers, as they may either silence genes that prevent tumor growth or activate oncogenic pathways. Their ability to influence multiple cellular processes makes them key players in cancer progression and potential targets for therapeutic intervention (50).

The regulatory effect of miRNA on the *B7-H3* gene is evident in several cancers, including osteosarcoma, clear cell renal carcinoma, multiple myeloma, medulloblastoma, mantle cell lymphoma, neuroblastoma, ovarian, colorectal and cervical cancer. Several miRNAs, such as miR-199a, miR-128 and miR-187, bind to the 3’UTR region of B7-H3 and regulate its expression (51–53). In neuroblastoma, it was found that depletion of miR-29 (miR-29a, miR-29b, and miR-29c) was associated with poorer patient survival. Experiments showed that these miRNAs are responsible for degrading B7-H3 mRNA and activating NK cells (54). Using TCGA data analysis, researchers found that decreased levels of miRNA 29c were linked to poorer survival in quadruple negative breast cancer (55). In head and neck squamous cell carcinoma, downregulation of miR-214-3p inhibited CD8^+^ T cell activity and facilitated disease progression (56). miR-34a has been shown to drive immune evasion in CRC cells by inhibiting SIRT1 and inducing B7-H3 and TNF-α in the tumor microenvironment (57). Similarly, miR187 was found to inhibit the growth and invasion of CRC cells by targeting B7-H3. This miRNA was downregulated in CRC compared to controls and was associated with shorter overall survival (53). In CRC, upregulation of miR-155 inhibits miR-143, leading to higher B7-H3 expression and the activation of T cells to release TGFβ, which promotes tumor growth (58). miR-128 was also found to downregulate B7-H3, which is typically highly expressed in CRC cells. This miRNA represses CRC migration and proliferation (59). Upregulation of miR-29c in CRC cells reduced B7-H3 expression, inhibiting cancer progression, invasion and migration (60). Similar findings were observed in ovarian cancer, where higher miR-29c expression downregulated B7-H3 and activated NK cells (61). In cervical cancer, higher expression of miR-199a was associated with inhibition of tumor migration, proliferation and invasion by targeting B7-H3. This effect was mediated through the AKT/mTor signaling pathway (62). In lung cancer, lower miR-145 expression was correlated with higher B7-H3 levels and increased lymph node metastasis (63). In medulloblastoma, reduced expression of miR-1253 resulted in increased B7-H3 expression, which promoted tumor cell proliferation and aggressiveness. miR-1253 was found to reduce tumor progression by arresting cells at G_0_/G_1_ phase of the cell cycle (64). In mantle cell lymphoma, increased miR-506 expression decreased B7-H3 levels, inhibiting cancer cell proliferation and metastasis (65). Nygren et al. comprehensively analyzed miRNAs regulating B7-H3 in breast cancer. They identified thirteen miRNAs that target B7-H3 directly by binding to the 3′-UTR region: miR-214, miR-363*, miR-326, miR-940, miR-29c, miR-665, miR-34b*, miR-708, miR-601, miR-124a, miR-380-5p, miR-885-3p, and miR-593. Moreover, miR-29c was related to significantly reduced risk of developing metastasis and had lower expression in higher grade tumors (33). Overall, the higher expression of these miRNAs in various cancers reduces B7-H3 levels, thereby inhibiting tumor growth. These findings provide a solid reason for developing more effective combination therapies that utilize miRNAs to target B7-H3 across different cancers.

## 5 Mechanism of action and role in cancer

**Figure 1:**
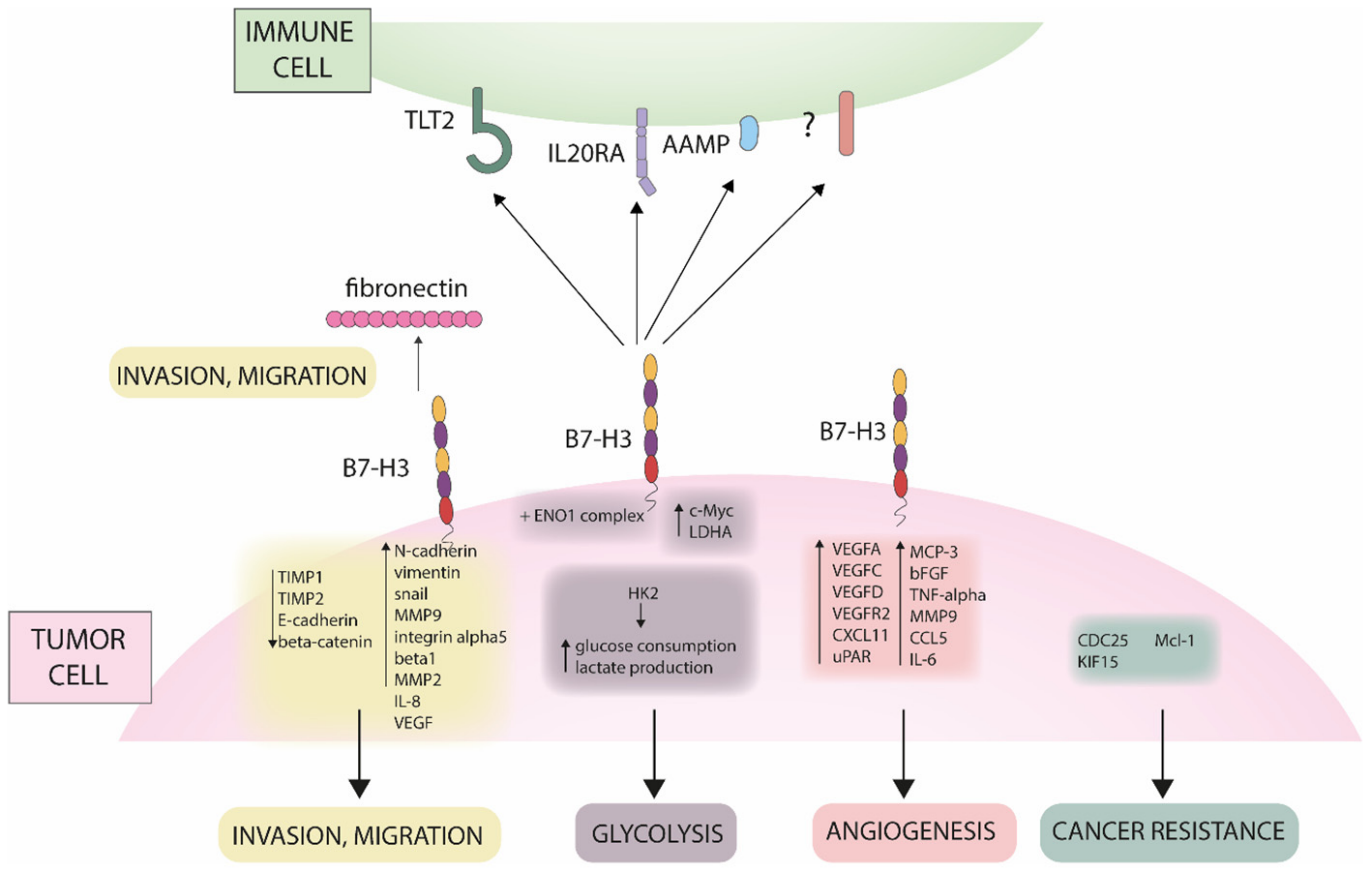
Different role of B7-H3 in cancer. The figure shows mechanisms of B7-H3 in promoting invasion, migration, glycolysis, angiogenesis and cancer resistance.

**Figure 2:**
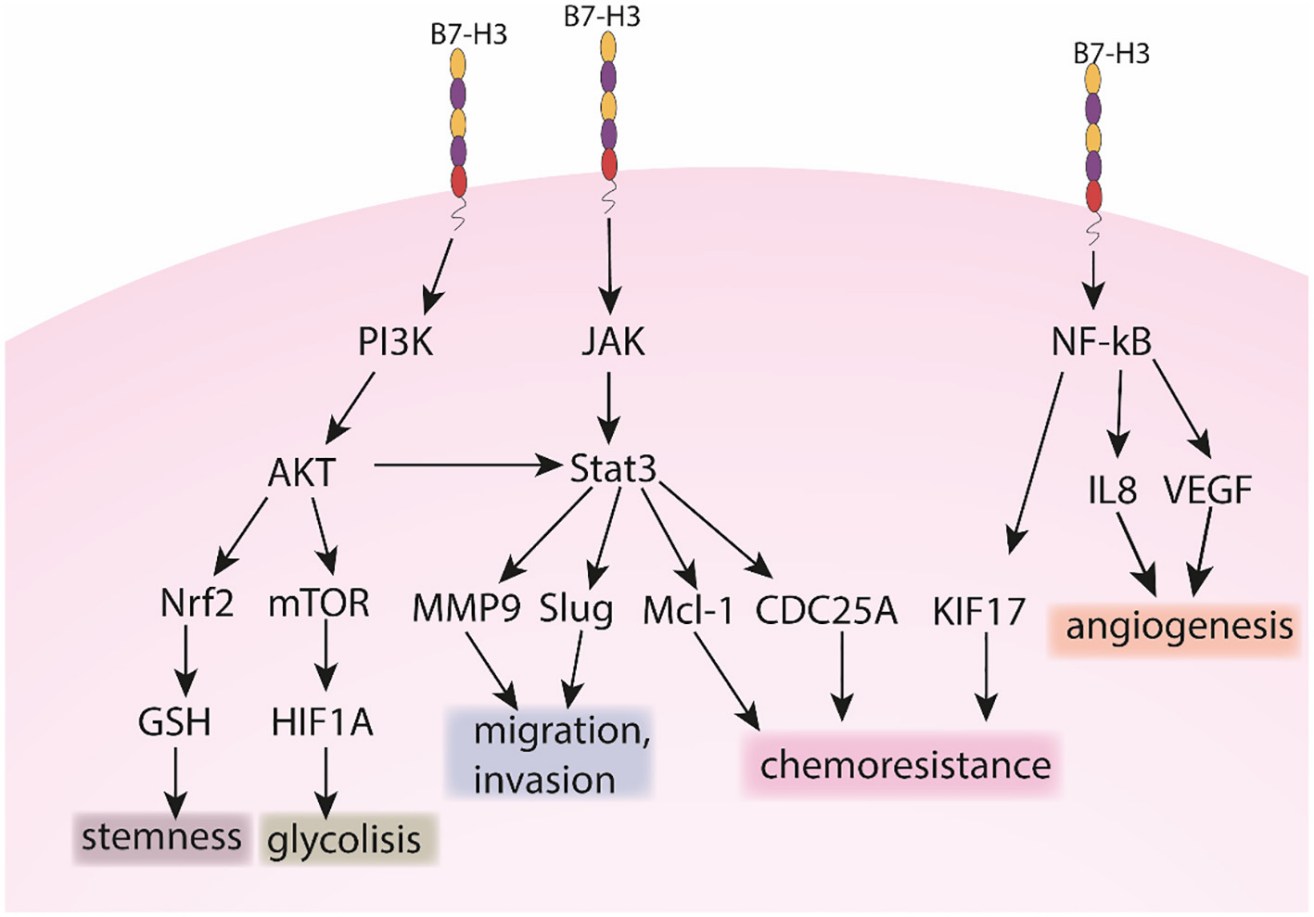
The role of B7-H3 in PI3K, JAK and NK-kB pathways.

Although B7-H3 is not an established cancer biomarker, lately it started to gain importance because of its overexpression in cancer compared to healthy tissues (66). B7-H3 is correlated to several cancer-related processes such as metabolism, angiogenesis, invasion and therapy resistance (67), including via JAK/STAT, NK-κb and PI3K pathways as summarized and illustrated in **Figures 1**, **2**. B7-H3 is expressed in different cells in the tumor microenvironment such as cancer cells, cancer stem cells, dendritic cells, pro-tumor type 2 macrophages, myeloid-derived suppressor cells, monocytes, endothelial cells (ECs), NK cells and cancer-associated fibroblasts which indicates the extensive engagement of the B7-H3 ligand/receptor interaction within the tumor region (67).

### 5.1 B7-H3 expression in cancer

**Figure 3:**
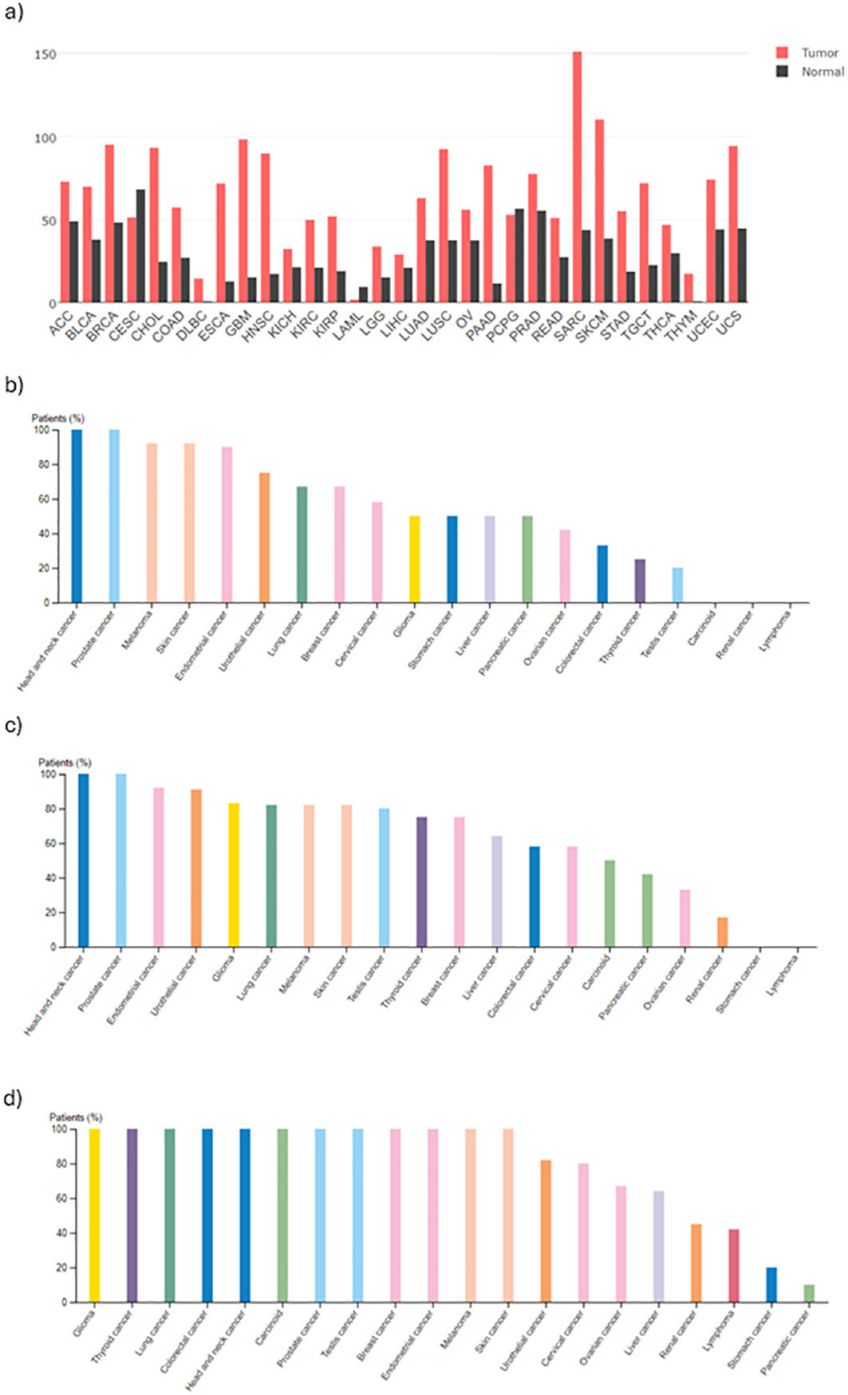
Summary of B7-H3 expression levels in different cancers. **(A)** mRNA expression in different cancers, obtained from GEPIA (71). Images of IHC staining obtained from the Protein Atlas (https://www.proteinatlas.org/) using three different antibodies **(B)** HPA009285, **(C)** HPA017139, **(D)** CAB017826.

B7-H3 is highly expressed in several cancers (**Figure 2**). Zhang et al. reported B7-H3 expression in 93% of analyzed ovarian tumor samples (96/103), where the protein presented with membrane and cytoplasmic localization (68). In contrary, B7-H3 expression was not detected in non-neoplastic ovarian specimens. They also reported that high B7-H3 expression was positively correlated to increased recurrence and mortality. The results of their study also show association between B7-H3 expression in tumor vasculature and histological type, stage, recurrence incidence, and poor clinical outcome. MacGregor et al. used B7-H3 as a novel target to be used in combination with existing therapies to overcome immunosuppression in the TME of ovarian cancer cells (69). With immunohistochemistry (IHC), the authors showed that B7-H3 is expressed by both tumor and stromal cells in the epithelial ovarian TME. In flow cytometry, both tumor and stromal cells were positive for surface B7-H3 expression which was lower in tumor cells compared to stromal cells. Analysis of TCGA dataset showed that B7-H3 expression is positively correlated to stromal markers (fibroblast activation protein alpha (FAP) and platelet-derived growth factor receptor beta (PDGFRβ)) and negatively correlated to epithelial markers (EpCAM and E-Cadherin). Since B7-H3 was found to be broadly expressed on tumor cells, stromal cells, and antigen-presenting cells (APCs), the authors suggest that B7-H3 therapies can target multiple cell populations. However, because its expression on non-tumor cells can be induced under specific conditions, possible off-target effects and toxicities should be considered beforehand. Yonesaka et al. reported that 74% of the examined non-small cell lung cancer (NSCLC) tested positive for B7-H3 in IHC while normal lung cancer was not stained (70). They also examined responsiveness to anti-programmed cell death protein 1 (PD-1) therapy and B7-H3 expression levels and reported that patients whose tissues were not stained for B7-H3 benefited more from anti-PD-1 therapy than patients whose tissues stained positive for B7-H3. The authors suggest that besides correlated to poor survival, high B7-H3 expression may be correlated to refractoriness to anti-PD-1/programmed death-ligand 1 (PD-L1) immunotherapy by impairing CD8+ T-cell mediated tumor immunity. **Figure 3A** (72) shows mRNA expression of B7-H3 across different tumors *versus* normal tissue. In all tumors, except for three, the expression is higher in tumor *versus* normal tissue. Those three exceptions are cervical squamous cell carcinoma, acute myeloid leukemia, and pheochromocytoma and paraganglioma. The protein expression (**Figures 3B–D**) show that B7-H3 is highly expressed in glioblastoma also on protein level.

### 5.2 The dual role of B7-H3 in immune system regulation

B7-H3 was initially thought to be a T-cell stimulating protein, but more recent studies show that it acts as a T-cell inhibitor (73). One of the first studies to investigate the role of B7-H3 showed that it increases the proliferation of CD4+ and CD8+ T cells and is crucial for interferon‐gamma (IFN‐γ) formation during T cell activation. In mouse models, it was also proved to activate tumor-specific cytotoxic T cells and enhances antitumor immunity. However, another study showed that B7-H3 plays a co-inhibitory role, inhibiting the proliferation of CD4+ and CD8+ T cells and reducing IL-2 and IFN-γ levels. It has also been confirmed to inhibit NK cell activity and promote osteoblast differentiation (74). Oh et al. investigated the role of B7-H3 in osteoclast differentiation. B7-H3 is highly expressed in osteoclasts and its inhibition leads to inhibition of osteoclastogenesis as well as increased IFN signaling, signal transducer and activator of transcription 1 (STAT1) activation and indoleamine 2,3-dioxygenase (IDO) induction as a downstream mechanism (75). Zhang et al. analyzed the role of B7-H3 in children with allergic asthma. They found that children with asthma had higher levels of B7-H3 compared to control subjects. They also showed that B7-H3 is a target of miR-29c and is regulated by Th2/Th17 cell differentiation (76).

In cancer, Sun et al. have shown that overexpression of B7-H3 leads to a complete regression of 50% of lymphomas and significantly reduces tumor growth in mice. In addition, the mice acquired systemic immunity against tumor cells and antitumor immunity was mediated by CD8+ T cells and NK cells (77). Similarly, Muo et al. investigated the effect of overexpression of B7-H3 in mouse mastrocytomas. Transfection with the gene resulted in enhanced immunogenicity, which led to tumor regression. It also induced a clonal expansion of CD8+ cytotoxic T lymphocytes (CTL) (78). In human studies, Loos et al. observed that pancreatic cancer patients with high levels of B7-H3 had a better prognosis than patients with low levels. Furthermore, the expression correlated with the number of CD8+ T cells (79). Similarly, Wu et al. analyzed the expression of B7-H3 in patients with gastric cancer. Higher expression of B7-H3 in the tissues was associated with better survival (80). In contrary, several other studies have shown that higher expression of B7-H3 in cancer is associated with poorer survival and disease progression, such as in prostate cancer and NSCLC (23, 79, 81–85). As Suh et al. have shown, B7-H3 inhibits the proliferation of T cells, preferentially downregulating type 1 cells rather than type 2 cells (86). It has also been observed that B7-H3 is associated with a reduction of IFN-γ and TNF-α. These are known to be produced by activated T cells and are toxic to tumor cells (87). B7-H3 mediates the immunosuppression via CCL2-CCR2 axis, which leads to M2 macrophage migration and differentiation as observed in ovarian cancer (88). B7-H3 has also been investigated in inflammatory diseases, such as arthritis. Yang et al. discovered that there is positive correlation between symptoms severity and B7-H3 expression on macrophages. The activity of B7-H3 is via NF-kB pathway. When mice with arthritis were treated with anti-B7-H3 antibody, the mice had weaker symptoms, and the inflammation reduced (89).

### 5.3 Role of B7-H3 in metastasis

Beside the immunological role, B7-H3 has an important role in tumor metastasis, as observed in several studies. Chen et al. investigated the role of B7-H3 in invasion. Downregulation of the gene led to reduced cell adhesion to fibronectin and reduced migration and invasion in melanoma and breast cancer cells. On the other hand, it played no role in proliferation (90). Similarly, in prostate cancer cells, Yuan et al. observed that downregulation of B7-H3 had no effect on cell proliferation. Also, B7-H3 blocks the interaction of cells with fibronectin and is related to increased cell migration and invasion (85). On the other hand, Yu et al. observed that B7-H3 promotes proliferation of lung cancer cell lines and similarly promotes invasion and migration. They also analyzed the expression of important epithelial and mesenchymal markers. After siRNA transfection, the levels of N-cadherin, vimentin and Snail were reduced, while the expression of E-cadherin was increased (91). Xie et al. investigated the role of B7-H3 in clear cell renal cell carcinoma. Knockdown of the gene led to a decrease in N-cadherin, MMP9, integrin alpha5 and beta1 genes. They showed that fibronectin promotes migration and invasion, but this effect is diminished in cells that have the B7-H3 gene knocked down. Their results also suggest that B7-H3 forms a complex with exogenous fibronectin (92). Furthermore, Xie analyzed the mechanism behind the B7-H3 related invasion in pancreatic cancer cells. sB7-H3 induced metastasis and invasion of cancer cells. It first upregulated Toll-like receptor 4 (TLR4) expression and then activated nuclear factor kappa B (NF-κB) pathway. This in turn increased the expression of IL-8 and vascular endothelial growth factor (VEGF) (93). Tekle et al. published a comprehensive study on the role of B7-H3 in melanoma cells. Similar as in previous studies, silencing of B7-H3 reduced migration and invasion *in vitro*. The effect was also translated *in vivo*, as silencing reduced metastatic potential and symptoms-free survival in mice and rats. The possible mechanism behind the metastatic potential of B7-H3 was also analyzed. In cells with knockdown, metastasis-associated proteins such as MMP2, Stat3 and IL-8 were decreased, while TIMP metallopeptidase inhibitor 1 (TIMP1) and TIMP metallopeptidase inhibitor 2 (TIMP2) were increased (94). In hepatocellular carcinoma (HCC) B7-H3 promotes cell invasion by targeting epithelial-mesenchymal transition (EMT) via partially activating JAK2/STAT3/Slug signaling pathway (95). Kang et al. first examined B7-H3 expression in HCC and found positive correlation between high B7-H3 expression and HCC metastasis. Moreover, increased B7-H3 intensity level was detected in metastatic HCC compared to other non-metastatic primary HCCs. Therefore, high B7-H3 expression was correlated to aggressive and metastatic HCC consequently also with cell migration. The authors reported that B7-H3 downregulation significantly reduces cell migration and matrigel invasion and has no effect on cell proliferation or apoptosis. In their study EMT-related protein E-cadherin was upregulated, while N-cadherin and vimentin were downregulated in B7-H3 silenced cells compared to negative controls. They propose B7-H3 to be used as a marker for tumor recurrence and/or metastasis. In a different study, Li et al. showed that silencing B7-H3 significantly suppressed migration and inhibited proliferation of lung cancer cells A549 and H460 compared to negative controls (96). Moreover, they reported that knock down of B7-H3 downregulated the expression of many integrin-associated proteins. In CRC, B7-H3 also may promote EMT by decreasing expression levels of E-cadherin and β-catenin and increasing expression levels of N-cadherin and vimentin (97). Liu et al. investigated the role of B7-H3 in the migration and invasion of CRC cells (98). With *in vitro* wound healing and transwell assays they observed that enhanced expression of B7-H3 promotes cell migration and invasion, respectively. The related pathway identified was Jak2/Stat3 which is known to be important in cell migration, invasion and metastasis. The authors reported MMP-9 to be a downstream target of B7-H3. Overexpression of B7-H3 increased phosphorylation of Jak2 and Stat3, which resulted in increased expression of MMP-9. B7-H3 is upregulated in bladder cancer where it promotes cell migration and invasion through the phosphoinositide 3-kinases (PI3K)/protein kinase B (AKT)/STAT3 signaling pathway (99).

### 5.4 Role of B7-H3 in glycolysis

As observed in several studies, B7-H3 has an important role in metabolism, particularly glycolysis. Nunes-Xavier analyzed the effect of B7-H3 levels in triple-negative breast cancer cell lines on sensitivity to 22 different anticancer drugs. In cells with B7-H3 knocked out, apoptosis inhibitor gene (API-2) and everolimus, which target the PI3K/AKT/mammalian target of rapamycin (mTOR) signaling pathway, showed greater inhibition of cell viability. In cells with B7-H3 knockdown, glycolytic capacity was reduced, while cells with B7-H3 overexpression exhibited higher glycolytic activity. Similar to previously observed, overexpression of B7-H3 had a weak effect on cell proliferation (100). Shi et al. investigated the role of B7-H3 in the metabolism in CRC cells. B7-H3 promotes glucose consumption and lactate production through the expression of hexokinase 2. In addition, B7-H3 also increased chemoresistance in cancer cells via hexokinase 2 (70). Li et al. similarly analyzed the effect of B7-H3 in oral squamous cell carcinoma. Elimination of B7-H3 resulted in decreased proliferation, colony formation, migration and invasion of the cells. B7-H3 also promoted glycolysis by regulating HIF1A hypoxia inducible factor 1 subunit alpha (HIFα) *via* the PI3K/AKT/mTOR pathway (101). Another mechanism was proposed by Zuo et al., who analyzed the role of B7-H3 in cervical cancer. Knockdown of B7-H3 resulted in decreased cell proliferation in the HeLa cell line. They also observed that B7-H3 forms a complex with the enolase 1 (ENO1) protein, confirmed by liquid chromatography–mass spectrometry (LC-MS) and IP. ENO1 is an important enzyme in glycolysis, and when the level of B7-H3 was reduced, adenosine triphosphate (ATP) and lactate production were also reduced. In addition, cellular Myc (c-Myc) and lactate dehydrogenase A (LDHA) were also reduced (102).

### 5.5 Role of B7-H3 in cancer resistance

B7-H3 has a significant role in cancer resistance to both, chemo- and radiotherapy. Ma et al. revealed that patients with low expression levels of B7-H3 presented with better overall survival compared to patients with high B7-H3 expression levels. It is reported that B7-H3 enhances chemoresistance of CRC cells by regulating the expression of cell division cycle 25A (CDC25A) through STAT3 signaling pathway in CRC cells (103). Moreover, overexpression of B7-H3 enhanced chemoresistance by reducing the G2/M phase arrest in a CDC25A-dependent manner. While the overexpression of B7-H3 promoted CRC cell viability and colony formation, chemotherapy-induced apoptosis was significantly decreased in B7-H3-overexpressing CRC cells *in vitro* and *in vivo*. Chemotherapy sensitivity was increased in cells with a stable knockdown of B7-H3. Ma et al. conclude that CRC cells can acquire chemoresistance through the B7-H3/CDC25A axis. In breast cancer, high expression levels of B7-H3 are correlated to poor outcome and resistance to commonly used chemotherapeutics such as paclitaxel (104). To study the role of B7-H3 in the sensitivity of metastatic breast cancer cells to paclitaxel, Liu et al. silenced B7-H3 in three breast cancer cell lines (MDA-MB-231, MDAMB-435, and MDA-MB-436). They reported that silencing B7-H3 enhanced the effect of paclitaxel chemotherapy compared to parental and control cells. Silencing B7-H3 abrogated the phosphorylation of Stat3 *via* inactivation of Jak2 and downregulated the direct target genes and anti-apoptotic factors induced myeloid leukemia cell differentiation protein (Mcl-1) and, to a lesser extent, survivin. On the contrary, its overexpression increased the phosphorylation of Jak2 and Stat3. *In vitro* results were also confirmed *in vivo* where the growth of B7-H3 knockdown xenografts was significantly inhibited upon paclitaxel treatment, while control tumors were only marginally affected. They concluded that Jak2/Stat3 pathway contributes to B7-H3–mediated paclitaxel resistance. Improving the sensitivity to paclitaxel by silencing B7-H3 is an important step into the clinical management of (metastatic) breast cancer. In CRC, B7-H3 enhanced the resistance to irradiation through upregulation of Kinesin family member 15 (KIF15) expression via NF-κβ which activated the extracellular signal-regulated kinase 1/2 (ERK1/2) pathway (105). Moreover, in CRC B7-H3 has an inducible effect on the polarization of macrophages from anti-tumor M1 into pro-tumor M2 (106).

### 5.6 Role of B7-H3 in angiogenesis

Several studies show that B7-H3 promotes angiogenesis. Wang et al. investigated the role of B7-H3 in CRC. As observed *in vitro* and *in vivo*, knocking down B7-H3 inhibits tube formation in human umbilical vein endothelial cells and leads to decreased expression of VEGFA, VEGFC, confirmed at mRNA and protein levels. The expression of VEGFA was induced via NF-κB, indicating the mechanism behind the angiogenesis (107). Purvis et al. investigated the role of B7-H3 in medulloblastoma, the most common embryonal neuroepithelial tumor. B7-H3 promoted angiogenesis by stimulating the secretion of VEGF. Afterwards, the conditioned medium of cells with B7-H3 overexpression was analyzed. Several pro-angiogenic factors were increased, including IL-6, IL-1, VEGF-D, VEGFR2, C-X-C motif chemokine ligand 11 (CXCL11), urokinase plasminogen activator surface receptor (uPAR), monocyte-chemotactic protein 3 (MCP-3), basic fibroblast growth factor (bFGF), tumor necrosis factor-alpha (TNF-α) and MMP-9, and chemokine (C-C motif) ligand 5 (CCL5 or RANTES) (108). In NSCLC Fan et al. investigated the role of B7-H3 in angiogenesis and alternative microvascular circulation, which is independent of angiogenesis, vascular mimicry. Cancer cell with B7-H3 knockdown resulted in decreased expression of E-cadherin and MMP-14, while there was no change in VEGF secretion. In the 3D model with B7-H3 knockdown, tumor growth was significantly reduced and the formation of capillary-like tubular structures was decreased. In the *in vivo* xenograft, B7-H3 knockdown resulted in significantly reduced tumor growth and decreased formation of vascular mimicry, while there were no changes in CD31+ endothelial vessels. Analysis of the supernatant of the cultured cells revealed no change in VEGF production between knockdown and mock cells. The proposed signaling pathway regulating vascular mimicry is via PI3K/AKT (109). Son et al. investigated the role of B7-H3 in angiogenesis. Suppression of B7-H3 resulted in decreased proliferation, increased apoptosis, and decreased migration of late endothelial progenitor cells, which are important for recovery from vascular dysfunction. However, this also led to increased tube formation and angiogenesis. This suggests that B7-H3 is necessary to maintain the cell population while blocking the promoted angiogenic differentiation (110). Lai et al. also showed that B7-H3 promotes VEGF secretion, that leads to angiogenesis (111). Seaman et al. conducted a comprehensive study on B7-H3’s role in promoting angiogenesis. Their findings revealed high expression levels of B7-H3 on both cancer cells and tumor-associated blood vessels. Leveraging this expression pattern, they developed an anti-CD276 drug conjugate, effectively targeting both tumor cells and infiltrating blood vessels. This dual-targeting approach highlights B7-H3 as a significant therapeutic target for inhibiting tumor growth and vascular support within the tumor microenvironment (66).

### 5.7 B7-H3 in cancer stem cells

Several studies show that B7-H3 is associated with cancer stem cells. For example, Liu et al. have shown that B7-H3 is upregulated in breast cancer stem cells and its inhibition leads to inhibition of cancer stem cells, both *in vitro* and *in vivo*. Namely, B7-H3 binds to major vault protein (MVP) and activates MAPK/ERK kinase (MEK) and the MAPK kinase signaling pathway (112). A similar mechanism was observed in prostate cancer, where B7-H3 is overexpressed in cancer stem cells (113). B7-H3 is also overexpressed in cancer stem cells in head and neck squamous cell carcinomas and is associated with the escape of anti-tumor immunity. Anti-B7-H3 antibodies have been observed to eliminate cancer stem cells, inhibit tumor growth and lymph node metastasis *in vivo*. The blockade also reduces EMT (114). Xia et al. conducted an in-depth analysis of B7-H3’s impact on stemness in gastric cancer stem cells, revealing that B7-H3 enhances this stemness through the AKT/Nrf2 pathway (115). This effect is mediated by B7-H3’s regulation of glutathione (GSH) synthesis, a critical factor in cellular redox balance that promotes cancer stem cell characteristics. Importantly, they demonstrated that inhibiting B7-H3 expression significantly reduces the stemness of these cancer cells, thereby suppressing tumorigenicity.

## 6 Role of B7-H3 in glioblastoma

There are reports that the expression level of B7-H3 in glioblastoma positively correlates to the malignancy grade and poor survival (116). Analyzing Chinese Glioma Genome Atlas (CGGA) and The Cancer Genome Atlas (TCGA) data, Zhang et el. reported high B7-H3 mRNA expression levels in isocitrate dehydrogenase (IDH)-wild type glioblastoma, which was correlated with the malignancy grade i.e. predicted significantly worse patient survival (21). The authors also analyzed the methylation pattern of B7-H3 and discovered that the B7-H3 gene promoter was significantly hypomethylated in CGGA and TCGA glioblastoma samples. In addition to this Wang et al. performed genetic and clinical characterization of B7-H3 expression using RNAseq data from CGGA and TCGA and confirmed high B7-H3 expression in high-grade gliomas (117). They also identified methylation of B7-H3 promoter and miRNA-29 as potential regulators of B7-H3 expression. Their gene set enrichment analysis (GSEA) network analysis revealed B7-H3 is highly correlated to mitotic cell cycle, cell proliferation, angiogenesis and upregulated immune response i.e. higher malignancy. Takashima et al. analyzed the expression of 67 immunotherapy-related genes involved in T-cell status as well as stimulatory and inhibitory checkpoint molecules in 571 non-treated primary glioblastoma patients. Their results suggested that the expression of a single gene *B7-H3* and its combination with *GATA3* and *LGALS3* are effective for glioblastoma prognosis (118). High expression levels of B7-H3 in glioblastoma were also confirmed by Nehama et al. (119). The authors analyzed B7-H3 mRNA expression levels in TCGA dataset and reported 77% of primary and recurrent glioblastoma samples presented with high expression. They also evaluated protein expression levels with IHC and observed strong staining in 76% (35/46) of analyzed cases. The authors noted that glioblastoma cells recruited around blood vessels were intensely positive for B7-H3. Furthermore, Nehama et al. generated B7-H3-redirected chimeric antigen receptor (CAR) (B7-H3.CAR) T cells encoding CD28 or 4-1BB endodomains and CD19-redirected CAR (CD19.CD28) T cells as control and tested them with U87MG and U138MG glioblastoma cells. In 5 days after seeding, the authors observed complete or near-complete elimination of U87MG and U138MG cells. Controls remained viable. At last, the antitumor activity of B7-H3.CAR-T cells was tested in a xenograft murine model. Tumor regression was observed in samples treated either with B7-H3.CD28 CAR-T cells or B7-H3.41BB CAR-T cells. Results were additionally confirmed using glioblastoma neurospheres in coculture and engrafted in mice. *In vivo*, B7-H3.CAR-T cells encoding either CD28 or 4-1BB equally controlled tumor growth and prolonged survival in 50% of treated mice as compared to controls.

In glioma, B7-H3 promotes EMT through activation of the JAK2/STAT3/Slug pathway. Zhong et al. examined B7-H3 protein expression levels in glioblastoma and lower grade glioma (LGG) tissues with IHC and found out that high B7-H3 expression levels are common in glioblastomas compared to LGG. They established B7-H3-overexpressing and knockout glioma cells to study the effect of B7-H3 on cell proliferation and invasive potential. Their *in vitro* and *in vivo* results showed that overexpression of B7-H3 promotes cell proliferation and invasion. In addition, they reported that B7-H3 induces EMT processes through downregulation of E-cadherin and upregulation of MMP-2 and MMP-9 expression via a JAK2/STAT3/Slug-dependent signaling pathway (120). In accordance with previously published data, knockdown of B7-H3 decreased while its overexpression increased the migration and invasion of glioblastoma cells as reported by Zhang et al. (121). In the same study, with RNA sequencing the authors examined transcriptome changes after B7-H3 knockdown and showed decreased expression of MYC proto-oncogene, BHLH transcription factor (MYC) and increased expression of SMAD family member 6 (SMAD6). Both genes are correlated to the TGF-β signaling pathways which was reported to be significantly enriched.

In glioblastoma both splicing variants, 2IgB7-H3 and 4IgB7-H3, of B7-H3 are present. In particular, 4IgB7-H3 is restricted to glioblastoma cells and may serve as a target for therapy, while 2IgB7-H3 presents with higher expression in recurrent glioblastoma and increases resistance to temozolomide (20). Therefore, 4IgB7-H3 can be further explored for therapeutic purposes whereas 2IgB7-H3 can be used to track tumor recurrence (122).

### 6.1 Distribution of B7-H3 and PD-L1 expression in brain cancer

**Figure 4:**
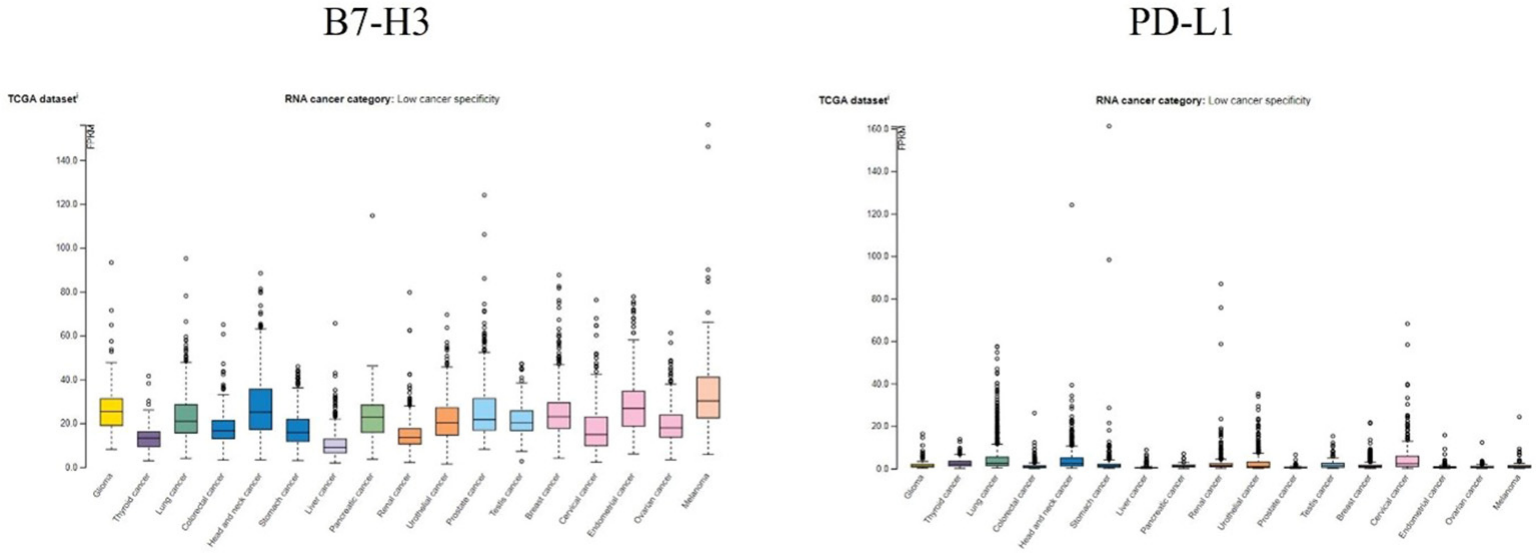
RNA expression overview of B7-H3 and PD-L1 in cancers analyzed in TCGA. FPKM, fragments per kilobase million. Data for B7-H3 and PD-L1 is from The Human Protein Atlas, access date 17 October 2024.

**Figure 5:**
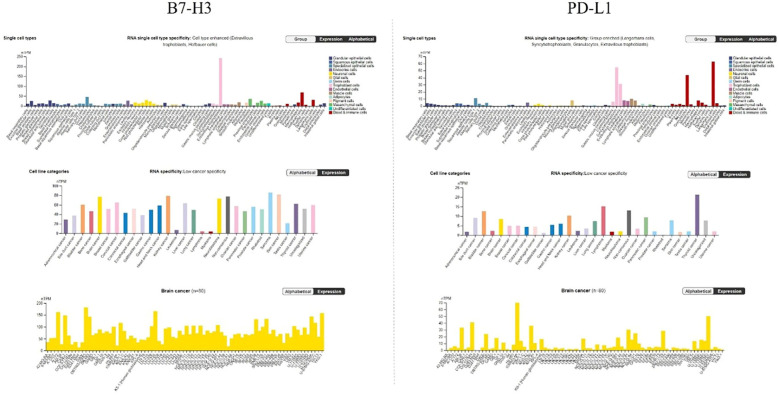
Expression levels of B7-H3 and PD-L1 in (top), RNA single cell type, (middle) different cancer cell lines, and (bottom) brain cancer cell lines. nTPM, normalized transcripts per million. Data for B7-H3 and PD-L1 is from The Human Protein Atlas, access date 17 October 2024.

The Human Protein Atlas (123) is a combination of transcriptomics and antibody-based proteomics aiming to map human proteins at a single cell and spatial resolution. It provides information about gene and protein expression levels in different reference and cancer samples. To contextualize the relevance of B7-H3 in brain cancers, we analyzed its expression pattern in brain cancer and compared it to the expression pattern of the well-known immune checkpoint PD-L1). Findings are presented in **Figures 4**, **5**.

In **Figure 4** RNA expression overview with data from TCGA is given. Expression levels of B7-H3 and PD-L1 were analyzed in 153 glioma samples and showed a median expression of 25.2 FPKM for B7-H3 and 1.2 FPKM for PD-L1. Both B7-H3 and PD-L1 have low cancer specificity.

Analyzing single cell types (**Figure 5**, top), in particular glial cells showed low expression of B7-H3 (6.2 astrocytes, 5.2 oligodendrocyte precursor cells, 1.5 oligodendrocytes, 7.2 microglial, 7.2 Muller glia, 1.9 Schwann cells) and PD-L1 (0.6 astrocytes, 0.9 oligodendrocyte precursor cells, 0.8 oligodendrocytes, 8.1 microglial, 0.7 Muller glia, 0.5 Schwann cells). When it comes to cell line categories, both B7-H3 and PD-L1 have low cancer specificity. Expression of B7-H3 and PD-L1 was analyzed in 80 brain cancer cell lines (**Figure 5**, middle) and showed a maximum average of 76.9 nTPM for B7-H3 and 8.5 nTPM for PD-L1. The more detailed analysis of expression of separate brain cancer cell lines (**Figure 5**, bottom) showed a maximum average expression of 181.7 nTPM for B7-H3 (cell line DBTRG-05MG) and a maximum average expression of 70 nTPM for PD-L1 (cell line IOMM-Lee).

### 6.2 Mechanisms of immune suppression of B7-H3 and PD-L1

Immuno surveillance refers to the processes by which host immune cells look for and recognize pathogens and altered cells such as cancer cells. Cancer cells however have developed various mechanisms to escape immune surveillance. The immunosuppression mechanisms of B7-H3 and PD-L1 are listed in **Table 1**.

**Table 1:** Mechanisms of immunosuppression of B7-H3 and PD-L1.

Immune checkpoint	Mechanism of suppression	Reference
B7-H3	B7-H3 expressed on APC or tumor cells recognizes a receptor on activated CD4+ and CD8+ cells	(26, 124)
	2Ig and 4Ig isoforms of B7-H3 inhibit the proliferation of T cells and downregulate cytokine production	(125)
	Promotes production of IL-10 and TGFβ to favor immunosuppressive environment	(126)
	Inhibits activity of CD4+ T cells, CD8+ T cells, γδT cells, CAR-T cells, Vδ2 T cells, Th17 cells, CD3+ T cells, NK cells, macrophages, neutrophils, dendritic cells, and secretion of IFN-γ, IL-2, perforin, granzyme B to favor immunosuppressive environment	(127–130)
	Promotes polarization of type 2 macrophages	(106)
	Contributes to CCL2–CCR2–M2 macrophage axis-mediated immunosuppression	(88)
PD-L1	Binds to PD-1 and exerts immunosuppressive regulatory effect via SHP2 by reducing the immune response of T cells	(131)
	PD-1/PD-L1 interaction: - suppresses T cells activation and proliferation, promoting T cell dysfunction and apoptosis - enhances function of T_reg_ and induces immune tolerance -promotes polarization of TAM and other immune cells into tumor-promoting phenotypes, facilitating immune escape and cancer progression	(132)
	Upregulation of PD-L1 and IL-10 expression in TAMs suppresses T cell proliferation and promotes tumor growth through the TLR4-MyD88-p38-STAT3 signaling pathway	(133)
	PD-1/PD-L1 axis inhibits NK cell-induced antitumor immunity *in vivo*	(134)

APC, antigen presenting cells; PD-1, programmed cell death protein 1; SHP2, Src homology region 2 domain-containing phosphatase-2; T_reg_, regulatory T cells; TAM, tumor associated macrophages; NK, natural killer cells

## 7 B7-H3 in cancer immunotherapy

**Figure 6:**
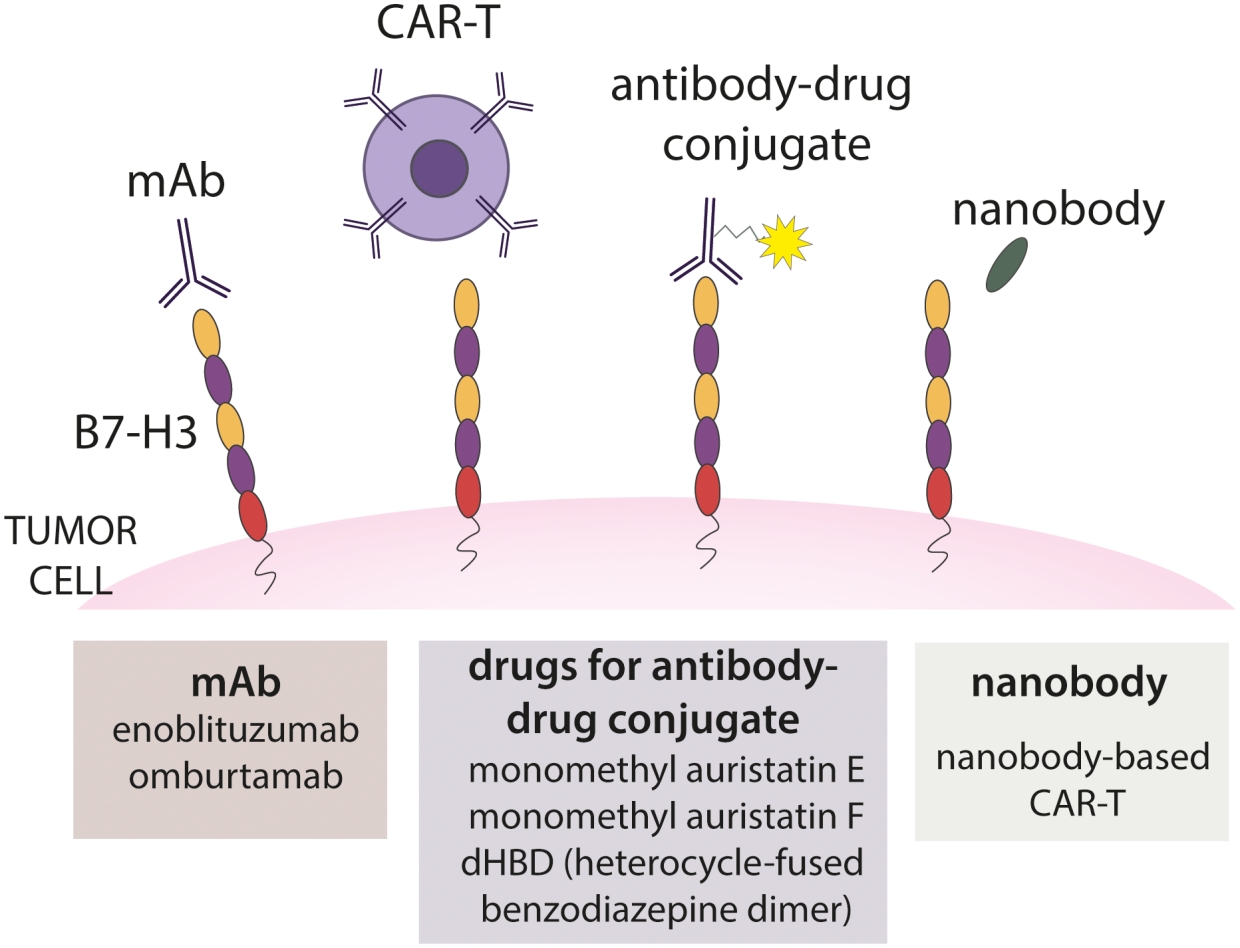
Different approaches of B7-H3 immunotherapy. Several approaches are presented, including targeting B7-H3 with monoclonal antibody, CAR-T, antibody-drug conjugate and nanobody.

Immunotherapy is a developing field in cancer treatment using human own immune system to recognize and destroy tumor cells. Different types of immunotherapies were developed. In the next section, various immunotherapeutic approaches based on B7-H3 which are illustrated in **Figure 6** will be explained with addition on using nanobodies as an approach to target tumors (30).

### 7.1 Monoclonal antibodies

Monoclonal antibodies are antibodies having only monovalent affinity for a single epitope. With changing their variable region, they can be designed to target any antigen or epitope (135). Therapeutic antibodies target cell surface antigens in cancers. The first developed monoclonal antibody for cancer treatment was against CD20 protein, which is highly expressed on cancerous B cells in non-Hodgkin lymphoma but not on healthy B cells (136). Until now, the WHO International Nonproprietary Names (INN) Program has assigned INN names to about 1,000 monoclonal antibodies, 530 of which are in the field of oncology (137). Success of monoclonal antibody in cancer treatment is now evident in multiple cancers including bladder cancer, sarcoma, colorectal cancer and breast cancer to name a few, targeting different proteins including those of B7-family PD-1 and PD-L1 (138).

Therapeutic monoclonal antibodies have different mechanisms of action: antibody-dependent-cellular-phagocytosis (ADCP), antibody-dependent-cellular-cytotoxicity (ADCC), complement-dependent cytotoxicity (CDC), radionucleotide delivery, cytotoxic drug delivery, neutralization, or signal blockade (139). The effect of the therapeutic monoclonal antibody is related to the antigen profile of the cancer, as well as the ability of the antibody to be internalized, activate Fcγ-receptors on innate cells, trigger activation of complement or block receptor-mediated oncogenic signaling (139). This depends on the isotope and nature of the monoclonal antibody (i.e., specific binding site, avidity of target binding, and particular conformation) and the target protein. Immune checkpoints enhance anti-tumor immune responses by restoring exhausted T cells. From the six major immune checkpoints for cancer immunotherapy, namely cytotoxic T-lymphocyte associated protein 4 (CTLA-4), programmed cell death 1 (PDCD1), cluster of differentiation 274 (CD274), inducible T-cell costimulator (ICOS), lymphocyte-activation gene 3 (LAG3), and CD40, ipilimumab targeted against CTLA-4 was the first monoclonal antibody approved by the Food and Drug Administration (FDA) in 2011. Ipilimumab activates the immune system by targeting CTLA-4, a protein receptor that downregulates the immune system, and is used in the treatment of metastatic melanoma. Ipilimumab mechanism of action is by blocking the binding of CTLA-4 to its ligands, inhibiting CTLA-4-mediated downregulation of T cells and promoting the interaction of CD80/CD86 with CD28 (137). This activates the immune system by increasing T cell expansion and enhancing the CTL-mediated anti-tumor immune response. Moreover, the IgG1-Fc region of ipilimumab binds to FcγRIIIa, induces ADCC and complement-dependent cytotoxicity (CDC) for enhanced anti-tumor efficacy by reducing T_reg_ cells.

Monoclonal antibodies have also been tested against B7-H3. In case of ovarian cancer cell lines treating with monoclonal antibodies alone significantly inhibited cell growth. In combination with tyrosine inhibitor even better outcome appeared (140). Shi et al. demonstrated monoclonal antibodies to inhibit 2IgB7-H3 and 4IgB7-H3 known to be highly expressed in variety of tumors (141). This success led to testing monoclonal antibodies including monoclonal antibodies against B7-H3 in clinical trials (**Table 2**). However, there are no clinical trials in phase 3 yet.

**Table 2:** Clinical trials targeting B7-H3 with monoclonal antibodies (142–147).

Trial number	Mechanism	Cancer	Stage	Goal	Result	Year
NCT02475213	Dual target of Enoblituzumab with Pembrolizumab or Retifanlimab	NSCLC Head and neck squamous cell carcinoma Urothelial cancer Melanoma	I/II	Outcomes and safety	Side effects in 87.2% of patients and grade ≥3 in 28.6% patients. One treatment-related death occurred (pneumonitis). Objective responses occurred in 33.3% patients with CPI-naïve HNSCC and in 35.7% patients with CPI-naïve NSCLC.	2022
NCT00089245	^131^I-Omburtamab	CNS malignancies	I	Efficacy and safety	In patients receiving cRIT for neuroblastoma, survival was markedly increased (median PFS 7.5 years) Acute toxicities included < grade 4 self-limited headache, vomiting or fever, and biochemical abnormalities. Grade 3/4 thrombocytopenia was the most common hematologic toxicity.	2022
NCT01502917	^124^I-Omburtamab	DIPG	I	Safety	One (4%) of 28 patients had treatment-related transient grade 3 hemiparesis and one (4%) had grade 3 skin infection. No treatment-related grade 4 adverse events or deaths occurred.	2018
NCT02982941	Enoblituzumab	Neuroblastoma Rhabdomyosarcoma Osteosarcoma Ewing sarcoma Wilms tumor Desmoplastic small round cell tumor	I	Safety	No results posted	2019
NCT02381314	Enoblituzumab plus ipilimumab	Melanoma NSCLC and other cancers	I	Safety	No results posted	2018
NCT01391143	Enoblituzumab	Refractory cancer expressing B7-H3	I	Safety	No results posted	2019

NSCLC, non-small cell lung cancer; HNSCC, CPI-naïve; CNS, central nervous system; cRIT -; DIGP, diffuse intrinsic pontine glioma; PFS, progression free survival.

For NCT02475213, patients with non-small cell lung cancer, head and neck squamous cell carcinoma, urothelial cancer and melanoma, were treated with enoblituzumab (investigational anti-B7H3 antibody), together with pembrolizumab, anti-PD1 inhibitor. 133 patients were enrolled in the study and 116 got treatment-related adverse effects, which were equal or treater than grade 3 in 28.6% of patients. There was also one treatment-related death due to pneumonitis. The response in HNSCC was in 6 cases out of 18 and in NSCLC was 5 of 14. The treatment was discontinued in 13 patients (148). Both NCT00089245 and NCT01502917, include therapy based on anti-B7-H3 antibody labeled with radioactive iodine. At clinical trial NCT00089245, 37 patients received injections of radiolabelled omburtamab. 16 of patients had metastatic neuroblastoma and other had solid tumors with B7H3 expression, including medulloblastoma, ependymoma, melanoma and rhabdomyosarcoma, choroid plexus carcinoma, atypical thabdoid teratoma, chordoma, pineoblastoma and retinoblastoma. The drug was overall well-tolerated. The most common adverse effect was myelosuppression. Patients who had neuroblastoma had significant improvement in overall survival and progression-free survival. Seven survived without progressive disease for 13-17 years from treatment. Of the remaining 8, only 2 had CNA relapse and the 6 died of systemic relapse or chemotherapy-related toxicity (149). At clinical trial NCT01502917, 28 children with diffuse intrinsic pontine glioma were treated with omburtamab labeled with radioactive 124-iodine (150). There were no dose-limiting toxicities. One out of 28 patients had treatment-related transient grade 3 hemiparesis and one had grade 3 skin infection. There were no treatment-related grade 4 adverse effects or deaths. The systemic exposure was low. The radioisotope was retained in the brain for more than 8 days.

### 7.2 Chimeric antigen receptor T cells (CAR-T cells)

CAR-T cell therapy is one of the main approaches of effective adoptive T-cell therapy (ACT), which kills cancer cells by the therapeutic use of transferred T cells (151). CAR is a synthetic construct i.e. a bioengineered receptor that binds to target cell surface antigens (e.g. proteins, glycolipids, and carbohydrates) through a single-chain variable fragment (scFv) recognition domain. CAR-T cells technology is an *ex vivo* method, which genetically modifies patient derived cells for immunotherapeutic purposes. Either NK cells or T cells can be used for CAR-T. Currently, various generations of CAR-T cells have been developed, each varying in their capacity to eliminate cancer cells (152). CAR-T cells mediate major histocompatibility complex (MHC)-unrestricted tumor cell killing by allowing T cells to bind to their cell surface antigens through a scFv recognition domain (151). Then, CAR-T cells form a non-classical immune synapse (IS), which is required for their effector function. These cells mediate their anti-tumor effects through perforin and granzyme axis, the Fas and Fas ligand axis, and cytokine release for sensitizing tumor stroma. The outputs largely depend on the individual components of the receptor i.e. scFv, spacer and costimulatory domains. First generation of CAR-T cell lacked co-stimulatory molecules which made them less powerful and inconsistent (153). However, newer generations of CAR-T cells added co-stimulatory molecules and improved its efficacy. Unlike conventional T-cells, CAR-T cells can bind to an antigen irrespective of the MHC presentation. However, their limitation is binding to cell surface expressed antigens only. CAR-T cells are currently used to treat hematological malignancies; namely, 6 CAR-T cell products have been approved by the FDA (154). Research for solid tumors is limited due to immunosuppressive TME and lack of antigens (152). Additionally, research on solid tumors was halted due to two deaths caused during clinical phase I in men with prostate cancer (155).

In clinical perspective, Majzner et al. tested B7-H3 CAR T cells on models of solid pediatric tumors (osteosarcoma, medulloblastoma and Ewing sarcoma) in xenograft mice (156). The authors developed a B7-H3 CAR, based on MGA271 (Enoblituzumab), that preferentially binds tumor tissues and shows restricted recognition of normal human tissues. They showed that the B7-H3 CAR T cells mediate antitumor activity *in vivo* and cause regression of established solid tumors. In particular, the B7-H3 CAR-T cells eradicated the autochthonous DAOY and D425 medulloblastoma xenografts which was observed by bioluminescent imaging. The systemically administrated B7-H3 CAR-T cells also mediated regression and eradication of established osteosarcoma and Ewing sarcoma xenografts.

CAR-T technology was tested for B7-H3 *in vitro* and *in vivo* experiments. Although no clinical trials have been completed so far, many clinical trials are currently recruiting participants. CAR-T cells targeting B7-H3 shows high tumor specific killing ability *in vitro* and tumor suppression effect *in vivo* in PDX (patient derived xenograft) model in osteosarcoma (157). CAR-T cells targeting B7-H3 are also effective in inhibiting growth *in vitro* and *in vivo* of NSCLC (158), prostate cancer (159), glioblastoma (116), ovarian and triple negative breast cancer (160).

### 7.3 Antibody drug conjugates (ADC)

Antibody drug conjugates (ADC) are a class of drug typically composed of monoclonal antibodies covalently attached to a cytotoxic drug i.e. payload and a linker in between. ADC combine the advantages of specific targeting ability and potent killing affect to accurately and efficiently eliminate cancer cells (161). Because of the antibody specificity, ADC target only antigen-expressing cells and therefore do not cause systemic toxicity which is common in conventional chemotherapy (148). When the antibody binds to the cell surface antigen on the target cell, the ADC is internalized to form an early endosome, followed by a maturation into late endosomes and finally fused with lysosomes. Linker breakdown promotes ADC release of the cytotoxic drug by chemical- or enzyme-mediated release into lysosomes which eventually leads to cell death or apoptosis via targeting DNA or microtubules (148, 161–163). Their mechanism of action is eliciting immunogenic cell death, ADCC, ADCP and CDC effects, as well as dendritic cell activation upon interaction with cancer and immune cells (161, 162). The “bystander effect” i.e. diffusion of the payload from antigen-expressing cancer cells to adjacent cells and therefore killing these cells contribute to the cytotoxicity of ADC in heterogenic tumors (148). Binding of the ADC to its target may also interrupt its downstream function by preventing the antigen interaction with its binding partners (163). ADC induce tumor-specific adaptive immunity by increasing the infiltration of T cells into the TME. Typically, they remain stable in blood, are target-specific and release cytotoxic component in the vicinity of cells. All components of ADC need to be carefully selected, including the right molecular target since they have an impact on safety, efficiency, and delivery of drug (161). Mostly used cytotoxic molecules inhibit tubulin, are immunomodulators or they damage DNA (164). Currently there are many ADC drugs that are under development for cancer treatment (161). First ADC drug granted approval by FDA was Mylotarg^®^ (gemtuzumab ozogamicin) for the treatment of adults with acute myeloid leukemia (AML) in 2000 (161, 165). The first ADC for treatment of solid tumors was Ado-Trastuzumab Emtansine (Kadcyla^®^) or T-DM1 for treatment of HER2+ metastatic or locally advanced breast cancer and was approved in 2013 (162). Recently two studies demonstrated efficacy of ADC targeting B7-H3 in glioblastoma (166), lung, and breast cancer cell lines and PDX models (167). Study using ADC for killing glioblastoma showed greater killing potency for glioblastoma cells with greater expression of B7-H3. They used monomethyl auristatin E which is known microtubule-disrupting agent. When coupled with fluorescent conjugate, they showed specific accumulation in tumor cells *in vivo* (166). ADC used for targeting B7-H3 in lung and breast cancer cell showed the same affinity as unconjugated B7-H3 antibody. This ADC cause cell cycle arrest in S phase, DNA damage and apoptosis. In this case, they used monomethyl auristatin F, which is also microtubule-disrupting agent (167). When it comes to ADC treatment, many of the patients develop resistance and experience disease progression. Therefore, the mechanisms of resistance (eg. altered target cell surface expression, gene mutations, changes in trafficking and internalization, payload resistance) need to be studied for optimal results. In addition, combining ADC with other chemo- or immune-therapeutic approaches may increase their utility in cancer treatment (163).

### 7.4 Antibody dependent cell mediated cytotoxicity (ADCC)

The immune mechanism of antibody-dependent cell-mediated cytotoxicity (ADCC) involves three components: effector cells with NK cells being the major type *in vivo*, antibodies and target cells opsonized by the antibodies (168). ADCC is a method of combating tumors by introducing specific antibodies that attach to target tumor cells. This attachment triggers or recruits effector immune cells to recognize these foreign antibodies and initiate cell death through non-phagocytic mechanisms. Antigens bind to the target cells through their antigen binding fragment (Fab), while the interaction with the effector cells occurs between the fragment crystallizable region (Fc) portion of the antibody (30, 168). There are three different mechanisms that occur after the activation of effector cells: cytotoxic granule release, Fas signaling, and elaboration of reactive oxygen species (ROS). From these, the main mechanism of action in ADCC is release of perforins and granzymes from effector cell granules. Activated effector cells can also upregulate the expression of Fas ligand in order to cause apoptosis in the target *via* Fas signaling. Although IgG, IgA, and IgE can mediate ADCC, IgG is the most used immunoglobulin subclass for cancer therapeutic antibodies. They developed humanized mouse antibody against B7-H3, which was afucosylated afterwards. Flow cytometry showed binding on IgC1 and IgC2 domain of human B7-H3. ADCC was activated against medium and high expressing breast (MDA-MB-231) and lung cancer (NCI-H322) cell lines. Afucosylated protein induced ADCC even on low expressing B7-H3 cancer cells, while non-afucosylated did not. Additionally, the afucosylated protein showed dose-dependent antitumor efficacy in severe combined immunodeficient (SCID) mouse and small effect in new generation of severely immunodeficient (NOG) mouse (169). Xu et al. developed bispecific modified protein targeting B7-H3/PD-L1 and with modified Fc region enhanced ADCC. Fusion protein blocked PD/PD-L1 pathway, activated CD8+ cells and enhanced ADCC in tumor cells with higher expression of B7-H3 (170). Another study found that B7-H3 human mouse chimera antibody induced ADCC in primary leukemia cells but not in hematopoetic cells. Furthermore, treating PDX models in combination with antibody and human NK cells significantly prolonged survival (171).

### 7.5 Small molecular inhibitors

Another important way of fighting cancer is using small molecular inhibitors. These molecules target different important molecules in signaling pathways of cancers including VEGFR, human epidermal growth factor receptor (HER), RAS, JAK, mTOR and so on. Because of their small size, small molecular inhibitors can bind to a wider range of extracellular and intracellular targets when compared to antibodies. Based on target specificity, small molecule inhibitors are divided into selective and multikinase. Multikinase small molecular inhibitors repress multiple kinases in the tumor, they do not require precise detection and rely on histology. Selective small molecule inhibitors bind to a single target and inhibit its cell signaling. They can either inhibit its unusual function or reverse its regular action. Selective small molecule inhibitors are then further divided into two groups: selective small molecule kinase inhibitors and selective small molecule non-kinase inhibitors (172). Selection of a small molecule whether it is a multikinase or selective small molecule kinase is based on the number of kinases whose inhibitory activity IC_50_ values are below 10 nM (173). Selective small molecule non-kinase inhibitors bind to targets beyond the kinome (i.e. the complete set of protein kinases encoded by the genome), thereby blocking subsequent functions to control tumors.

Protein kinase inhibitors are the main category of small molecule inhibitors. Protein kinase enzyme has important role in cell growth, proliferation and differentiation. Protein kinase catalyzes the transfer of γ-phosphate group from ATP to protein residues containing hydroxyl groups. Dysregulations of protein kinases are linked to various diseases including cancer. Because of this, protein kinases are studied as cancer therapeutic targets. The selective small molecule kinase inhibitors contain receptor-related kinase inhibitors, kinase inhibitors targeted intracellular signaling pathways, and inhibitors targeting other cytoplasmic kinases (172). As described in (174), protein kinase inhibitors are classified into six types: Type-I inhibitors bind to the active conformation of the kinase (DFG-Asp in, αC-helix in); type-I½ inhibitors bind to a DFG-Asp in inactive kinase conformation with αC-helix out; type-II inhibitors bind to a DFG-Asp out inactive conformation; type III inhibitors restrain kinase activity by binding to an allosteric site; type IV inhibitors bind outside of the cleft; type V inhibitors are bivalent molecules that span two distinct regions of the kinase domain; and type VI inhibitors that bind covalently with the kinase active site. While type I-V inhibitors are all reversible, type VI are irreversible kinase inhibitors. The tyrosine kinase inhibitor imatinib was the first small molecule targeted drug that was approved by the FDA in 2001 (174).

Small molecular inhibitors have some important advantages that can be readily used like pharmacokinetic properties, low manufacturing cost, drug storage and transportation, small size, short half-lives, patient compliance and good distribution in tissues (174, 175). Moreover, small molecule inhibitors can be taken orally, and some of them can penetrate the BBB to control intracranial lesions (172). However, they also present with disadvantages such as low response rate and drug resistance. Understanding that the receptor(s) on activated T cells interact with the FG loop of the IgV domain of B7-H3, there is potential to create a small molecule inhibitor aimed at disrupting this precise binding region. However, it is important to acknowledge that unforeseen off-target effects may occur, underscoring the need for comprehensive assessment to minimize any potential negative outcomes. To the best of our knowledge, there are currently no clinical trials targeting B7-H3 protein and none *in vitro* or *in vivo* studies regarding B7-H3.

### 7.6 Nanobodies

Nanobodies are ~15kDa proteins derived from heavy-chain only antibodies produced by just few animals, such as camelids and sharks. They were first discovered by the group of Raymond Hammers in Belgium at Vrije Universiteit Brussel in 1993 in camelids (176). Camelids have two types of IgG antibodies, the conventional and heavy chain antibodies that are lacking light chains. Nanobody is a variable domain of this heavy chain antibody. These nanobodies have several advantages compared to conventional antibodies. Due to their smaller size, they exhibit better tissue penetration and faster clearance from the body. Additionally, they are more stable due to several amino acids substitution that make them more hydrophilic. They contain 3 binding domains i.e. complementarity determining regions (CDRs - CDR1, CDR2, CDR3) compared to 6 in classical antibodies, but still bind with affinities in nano/pico molar concentrations (177). The longer CDR3 of nanobodies forms a finger-like extension that binds to small hidden epitopes in the concave surface of the antigen or in the antigen gap with high affinity. Nanobodies can selectively bind multifunctional epitopes on the receptor, block disease-related signaling pathways or responses (178). Zarantonello et al. described the selection of the C1q specific nanobody C1qNb75 that is able to inhibit activation of the classical pathway (CP) of the complement system (179). The proposed mechanism of action is by occluding the IgG binding site on C1q.

Nanobodies as the single agents have not been yet developed and tested as therapeutics. However, their unique and potentially buried binding sites, small size, as well as simple expression and production, make nanobodies suitable for use in CAR-T therapy development. Li et al. developed nanobody-based CAR-T cells targeting B7-H3 for treating several different solid tumors. Nanobody against B7-H3 was retrieved from dromedary camel phage library and specific CAR-T cells were obtained first by cloning nanobody into CAR construct. Human peripheral blood mononuclear cells (PBMCs) were then isolated from healthy donors and stimulated with anti-CD3/anti-CD28 antibody-coated beads in presence of IL-2. The therapy efficiently lysed four neuroblastoma (NB) cell lines, two pancreatic cancer cell lines, triple-negative breast cancer cell lines and lung adenocarcinoma cell line. CAR-T cells were tested also in mice model of pancreatic cancer, where they had high antitumor activity (180). One of the most important components in CAR-T cell technology is the antigen-recognition domain (180). B7-H3 has two distinct epitope domains IgC and IgV; one of the clinically tested antitumor antibodies, 8H9, binds to the IgV domain. In their study, Li et al. showed that the B7-H3 CAR-T cells targeting IgC are more potent than those targeting IgV in pancreatic ductal adenocarcinoma (PDAC) and NB mouse preclinical models. They conclude that the antigen-binding epitope is crucial for the biological function of antibody-based CAR-T cells.

## 8 Therapeutic approaches for glioblastoma and other brain tumors

**Table 3** below presents clinical trials targeting B7-H3 in glioblastoma patients. Currently there are five different clinical trials that are in phase of recruiting patients and all of them include CAR-T therapy. The majority of trials are Phase l.

**Table 3:** Clinical trials targeting B7-H3 in glioblastoma patients (181–186).

ClinicalTrials ID	Disease	Mechanism	Stage	Status
NCT04077866	Recurrent or refractory glioblastoma	Drug: Temozolomide Biological: B7-H3 CAR-T	Phase l/ll	Recruiting
NCT04385173	Recurrent or refractory glioblastoma	Drug: Temozolomide Biological: B7-H3 CAR-T	Phase l	Recruiting
NCT05241392	Recurrent glioblastoma	Biological: B7-H3-targeting CAR-T cells	Phase l	Recruiting
NCT05474378	Recurrent glioblastoma	Biological: B7-H3-targeting CAR-T cells	Phase l	Recruiting
NCT05366179	Recurrent or refractory glioblastoma	Biological: B7-H3-targeting CAR-T cells	Phase l	Recruiting

All clinical trials are designed for glioblastoma patients with recurrent and refractory glioblastoma. For three of the clinical trials, the inclusion criteria are the B7H3 expression in tumor, determined by immunohistochemistry. For NCT05241392, the expression of B7-H3 should be more or equal to 30%. For NCT04077866 and NCT04385173 the inclusion criteria positive B7-H3 positive tumor expression by immunohistochemistry at the initial tumor or recurrent disease with H-score more or equal to 50. For NCT05474378 and NCT05366179, there is no inclusion criteria for B7-H3 expression. There is no outcome for the trial listed.

A case study reported evaluation of the therapeutic potential of B7-H3 targeted CAR T-cell therapy in treating recurrent glioblastoma (187). A 56-year old woman presented with recurrent glioblastoma after two craniotomies and standard treatment with chemotherapy during the last 2 years. High B7-H3 expression levels were confirmed in primary patient cells using flow cytometry. B7-H3 targeted CAR-T cells induced specific anti-tumor effect in the primary cells. ELISA results also indicated an activation effect of the CAR-T cells when cocultured with tumor primary cells. After detecting tumor recurrence, the patient received weekly intracavitary infusions of B7-H3 targeted CAR-T cells with a dose-escalating principle. Tumor reduction was detected using magnetic resonance imaging (MRI) and the clinical response was maintained for approximately 50 days after the administration of B7-H3 targeting CAR-T cells. However, in cycles 6 and 7 the patient had another recurrence and dropped out of the clinical study. Headaches were reported as a side effect of the therapy starting from 3 h after infusion and increasing in follow up infusions. Headaches were probably as a result of inflammation response. The authors also detected a significant expansion of T cells in cerebrospinal fluid (CSF) samples obtained from the infusion device. In addition, they evaluated 16 inflammatory cytokines to assess immunologic changes in CSF and periphery blood before and after each cycle infusion. In particular, IL-2 and IL-6 levels increased significantly (factor >5) in periphery blood. The tumor became resistant to the treatment which may be a result of target antigen heterogeneity i.e. CAR-T cells were not able to completely eliminate the tumor cells especially those with low B7-H3 expression levels. Expansion of these cells may have led to tumor relapse.

Similarly, Tang et al. assessed bioactivity and safety levels of B7-H3 CAR-T cells against anaplastic meningioma (188). A 49-year old woman presented with multiple recurrent anaplastic meningiomas. IHC showed high and homogeneous expression levels of B7-H3, which was also confirmed with immunofluorescence staining of B7-H3 in primary cells from the patient tumor. Patient received 3 cycles of CAR-T cell treatment. Here, the patient also reported moderate headache as a side effect starting from 3 h after CAR-T cell infusion in 2^nd^ and 3^rd^ round. CAR-T cells were detected in CSF but were absent in peripheral blood. From the 6 inflammatory cytokines measured, only IL6 appeared with increased levels in the serum. This case study indicated that B7-H3-targeted CAR-T cells exhibited local antitumor responses against anaplastic meningioma. B7-H3 targeted CAR-T cells locally suppressed tumor progression without serious side effects which indicated the tolerability, safety and efficacy of the therapy.

Despite the promising early results, no B7-H3-targeting CAR-T cell therapies have been approved for clinical use.

## 9 Gaps in the current understanding of B7-H3 and future research directions

B7-H3 has gained attention in the fields of oncology and immunotherapy because of its expression pattern on tumor cells and safety profile (175). This also encouraged its potential use in therapeutic development, based on the knowledge and directions obtained from existing immunotherapeutic strategies involving other immune checkpoints such as PD-L1 and CTLA-4. Still, even with the progress in understanding the role of B7-H3 in cancer, there are several gaps in knowledge that remain to be filled. The molecular mechanisms by which B7-H3 contributes to cancer metastasis, angiogenesis and immune evasion can be elucidated by clarifying its exact intracellular signaling pathways and interactions with other immune checkpoints (e.g. PD-L1). In this regard, identifying the receptor of B7-H3 is crucial not only for elucidating its role in cancer, but also for understanding of its exact function and design of B7-H3 antagonists. The heterogeneity of B7-H3 expression within tumors and how it affects treatment as well as development of drug resistance should be explored in more detail. To evaluate its diagnostic and therapeutic potential, the expression profiles of B7-H3 in pre-malignant lesions, tumor-associated vasculature, metastases, recurrence as well as bodily fluids have to be determined. At last, development of suitable models for cancer modeling such as spheroids and organoids that will provide more information about the therapeutic potential of targeting B7-H3 alone and in combination with existing therapies (e.g. radio-, chemo- and immuno-therapy) will accelerate its translation into real clinical settings.

Two of the most extensively explored approaches are monoclonal antibodies and CAR-T cell therapy. The value of monoclonal antibodies in cancer treatment lies in their mechanism of action i.e. promoting cancer cell death by recognizing tumor associated antigens (TAA) on cancer cells, and stimulation of long-lasting antitumoral activities while leaving healthy cells untouched (189). Still, more than 20 years passed from the approval of the first monoclonal antibody for the treatment of autoimmune diseases, non-Hodgkin lymphoma and chronic lymphocytic leukemia (Rituximab, 1997) until the approval of the first antibody for treatment of solid tumors (Sacituzumab Govitecan, 2020). This can be a result of poor antibody penetration in tumor tissue and impaired homogeneous distribution, heterogeneous antigen expression on cancer cells and presence of immunosuppressive TME (190). Perhaps the combined use of multiple antibodies targeting various TAA will yield better results and improved patient outcomes. Bispecific antibodies that can simultaneously target TAA and activate T cells or other effector immune cells may also be beneficial (190). Moreover, novel antibodies and antibody classes targeting different antigens or different epitopes on existing and to-be-determined therapeutic targets should be explored (191). With such an approach more cancer cells will be destroyed which will leave little space for development of escape mechanisms. Changes in gene/protein expression levels during the course of treatment which can influence the therapeutic response should not be overlooked. Development of a new generation of antibodies with improved specificity, penetration and distribution, reduced off target effects and increased potency should follow. Similar to ADC the limitation of the monoclonal antibodies is development of therapy resistance which emphasizes the need to deepen the knowledge about their mechanisms of action. Because of their expensive production and complex nature strategies for cost reduction should also be proposed.

To date, 6 CAR-T cell therapies against hematological cancers are already approved by the FDA (180). However, although promising, the application of CAR-T therapy to solid tumors is not that remarkable and is limited by various factors such as target antigen heterogeneity, trafficking and a hostile TME. To address the problem of antigen loss, bispecific CAR-T cells may be used to avoid antigen escape relapse. Moreover, trafficking of the CAR-T cells is another problem as locally delivered CAR-T cells may not be able to reach distant tumors and target them effectively (188). In addition, low or heterogeneous antigen expression is suboptimal for efficient CAR-T cell therapy as it can result in development of treatment resistance and tumor relapse. On the other hand, low antigen levels i.e. below the threshold for effective CAR-T on non-malignant tissues may be tolerable (156). Related to this, the antibody on which CAR-T is based, MGA271, shows minimal binding to normal tissues (156). In early phase clinical trials MGA271 was proven safe, without major toxicities and resulted in meaningful responses (192).

## 10 Conclusion

The extensive development of immunotherapeutic approaches and their success in the clinic have prompted scientists to further improve current therapies and search for immunotherapeutic targets that have clinical potential. One of these is the transmembrane protein B7-H3 that is highly expressed in various types of cancer. Because of its multifaceted role in several key cancer processes like promotion of cell migration, invasion and proliferation, B7-H3 is an attractive target for cancer treatment. In regard to immunotherapy, several approaches have been developed to date, including CAR-T, ADC, ADCC and nanobodies, particularly their use in CAR-T. The novel immunotherapeutic approaches are highly sought after for problematic cancers for which there are no effective therapies. One of these is glioblastoma, a particularly deadly cancer with a low survival rate that has remained unchanged for years. Since higher B7-H3 expression levels are correlated to the malignancy grade and poor survival of glioblastoma patients it is worth exploring it as a biomarker for disease progression. The existence of both splicing variants in glioblastoma opens up new avenues for research and clinical translation. Namely, the specific expression of 4IgB7-H3 in glioblastoma cells and higher expression of 2IgB7-H3 in recurrent glioblastoma and its correlation to temozolomide resistance indicate that B7-H3 can be explored as a therapeutic target, but also as a biomarker of tumor recurrence. However, for better designing of targeted diagnostic and therapeutic approaches its receptor should be determined.
